# Community voices in health literacy: a qualitative exploration into perceptions of a health literacy mediator

**DOI:** 10.1093/heapro/daae130

**Published:** 2024-10-14

**Authors:** Madeline Spencer, Vaughan Cruickshank, Nenagh Kemp, Rosie Nash

**Affiliations:** School of Medicine, College of Health and Medicine, University of Tasmania, 17 Liverpool Street, Hobart, Tasmania 7001, Australia; School of Education, College of Arts, Law and Education, University of Tasmania, Newnham Drive, Newnham, Tasmania 7001, Australia; School of Psychological Sciences, College of Health and Medicine, University of Tasmania, Grosvenor Street, Sandy Bay, Tasmania 7001, Australia; School of Medicine, College of Health and Medicine, University of Tasmania, 17 Liverpool Street, Hobart, Tasmania 7001, Australia

**Keywords:** health literacy, health literacy mediator, interview, community, qualitative

## Abstract

Health literacy is a vital asset needed to empower individuals to take control of their health. An individual’s health literacy is the ability to find, use and apply health information and services to manage their health. They interact with the health services and members of their community who can offer additional support. Creating the role of a health literacy mediator (HLM) may help to improve health literacy outcomes for all. For this role to be accepted by individuals within a community, the community itself should be involved in the development of the roles and associated responsibilities. The aim of this study was to engage with community members to acquire their perspectives on the potential of this role. Qualitative semi-structured online interviews were used to engage in discussions with local community members. This study implemented a constructivist epistemology with qualitative research design. Data were thematically analysed to identify evolving themes that were important to the HLM role. The analysis identified three main themes that need to be considered when adopting an HLM role: (i) health empowerment of individuals, organizations and communities, (ii) meeting the needs of the community and (iii) addressing the existing barriers in navigating and accessing the healthcare system. Those working in the health promotion space must adopt novel and innovative ways to improve HL on both a local and an international scale. This study concluded that for the role of a HLM to be accepted, it would need to encompass these attributes.

**Contribution to Health Promotion**

- This study is the first to explore the concept of a health literacy mediator and how this role may look in practice. The co-design approach allows for the community voice to be brought in during the development phase of the role
- For the role to be accepted by the community and assist in enhancing health literacy it will need to empower, meet the needs and improve access to health for individuals, organizations and communities.
- Recommendations proposed here should be considered in future research, in the design, development and implementations of a health literacy mediator role.

## INTRODUCTION

Health equity is the state in which everyone has a fair and just opportunity to attain their highest level of health. However, health inequities can prevent individuals, families and communities from achieving this state (Braveman, 2014). These inequities may result from systematic differences in the health status of different population groups and can have significant social and economic costs to individuals and societies (World Health Organisation (WHO), 2018). Health inequity is regarded as a global crisis and ways to overcome such inequities can be found embedded in initiatives that aim to improve the health of the population. For example, within the Sustainable Development Goals (SDGs), a set of 17 global goals adopted by all United Nations Member States in 2015, ‘Good Health and Wellbeing’ (SDG3) and ‘Reduced Inequalities’ (SDG10), are acknowledged as vital to overcoming inequities in health and society (United Nations Department of Economic and Social Affairs (UN DESA), 2023).

The WHO recognizes that more needs to be done to promote progress to achieve the SDGs. To this end, in 2019, the WHO announced the Global Action Plan for Healthy Lives and Well-being for All (SDG3 GAP) (WHO, 2019). Its goal is to help countries accelerate advancement on the health-related SDGs and to offer support for equitable progress. The plan focuses on reinforcing sustainably financed primary health care, improving maternal and child health, preventing noncommunicable diseases and strengthening countries’ capacities to avert and respond to health crisis (WHO, 2019, 2023). A life-course approach to the SDGs aims to provide a conceptual framework for optimizing health and well-being, considering various individual, social and environmental factors that shape an individual’s health and well-being over time (Banati, 2018). This approach recognizes the interdependence of people, the society in which they live, and the policy and procedures surrounding them. This life-course approach can be employed to address critical, interdependent factors affecting health and sustainable development in a holistic manner (Kuruvilla *et al*., 2018). Challenges in implementing a life-course approach include meeting the need for good governance, appropriate policies and sustained improvements. This approach requires integrating individual, social, economic and environmental considerations, which can be complex (Kuruvilla *et al*., 2018). Furthermore, the implementation of a life-course approach necessitates a shared understanding of how health is shaped by various factors throughout life, which can be challenging to achieve.

Overall health is often influenced by non-medical elements that influence health outcomes. These encompass the circumstances of birth, growth, work and aging, alongside broader forces like economic policies, social norms, education, employment, housing and healthcare access (WHO, 2020). These factors are known as the social determinants of health (SDH). Recognizing and addressing SDHs is crucial for promoting health equity and enhancing overall population health. Health promotion increasingly aims to address the SDHs. By focusing on these factors, health promotion initiatives seek to reduce health disparities and inequities by tackling the root causes of poor health, such as poverty, discrimination and lack of access to resources (Artiga and Hinton, 2018). To achieve this aim and promote health equity, there must be contributions from a wide range of stakeholders within and beyond the health sector (Chelak and Chakole, 2023). While addressing all the SDHs at once may be an impossible mission, focusing efforts in on a specific SDH (health literacy) may positively impact health outcomes in communities.

**Fig. 1:**
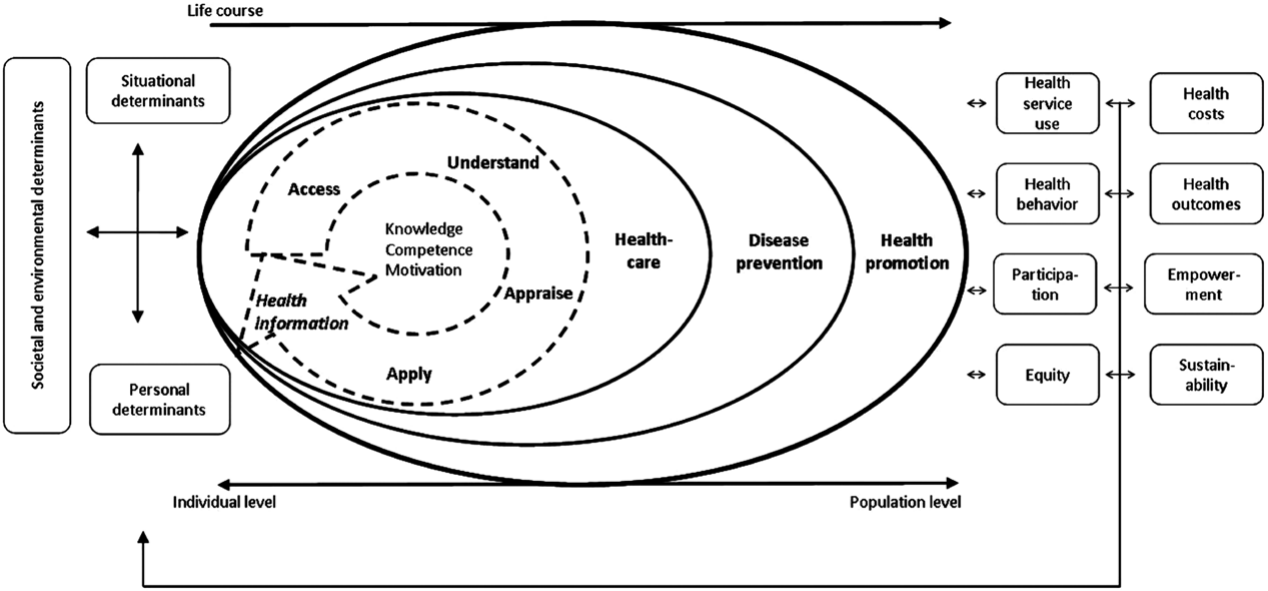
Integrated model of health literacy (Sørensen *et al.*, 2012. Originally published in BMC Public Health).

Health literacy (HL) is an important concept that can impact an individual’s overall health. It is also deemed to be a SDH in its own right (Marmot *et al*., 2008; Friis *et al*., 2016; Bröder *et al*., 2018). Sørensen *et al*. (2012, p. 3) defined HL as being *linked to literacy and entails people’s knowledge*, *motivation and competences to access*, *understand*, *appraise and apply health information in order to make judgments and take decisions in everyday life concerning healthcare*, *disease prevention and health promotion to maintain or improve quality of life during the life course*. The multifaceted nature of health literacy, which encompasses skills such as literacy, numeracy, verbal communication and the ability to navigate the healthcare system, poses challenges for researchers in developing standardized research methods and measurement tools. The research landscape is further complicated by the way that social, economic and cultural factors can differentially influence the impact of HL on health outcomes (Nutbeam, 2000; Guo *et al*., 2020). Thus, Sørensen *et al*. (2012) created and empirically validated a conceptual model outlining these intricacies, shown in Figure 1.

This model also shows how a life-course approach can be a framework for public health planning. Using this model can help in systematically addressing different domains of HL and in understanding how HL is influenced by the SDHs at different stages of life. Additionally, Sørensen’s work allows for the strategic targeting of HL promotion. Several studies and strategies have highlighted the importance of this approach, emphasizing an individuals’ role in actively managing their own health (Clouston *et al*., 2017; Guo *et al*., 2020; Maindal and Aagaard-Hansen, 2020). It should be acknowledged that not all individuals will be able to overcome the challenges they experience when accessing or navigating health information and services. This necessitates looking beyond the individual and examining the responsibility of health services and the community around those individuals.

International attention is now focused on ways to improve, adapt and develop the HL skills of individuals, organizations and communities. Some initiatives that have been theorized in the literature and implemented in practice include health navigators, health coaches and health advocates. These positions appear to perform similar roles, such as connecting people to medical providers and community resources, navigating the complexities of the healthcare system and overcoming individual barriers to care to achieve health goals (Natale-Pereira *et al*., 2011; Boroumand *et al*., 2020; Perlman and Abu Dabrh, 2020; Budde *et al*., 2022). Such roles have been implemented in practice across various countries including Australia, New Zealand and Canada, but are mainly found in the United States. They are situated in hospitals, clinics and private practices. Numerous studies demonstrate the positive impact of this type of role in providing personalized guidance, education and support. This can empower patients to better navigate the healthcare system, adopt healthy behaviours and access needed services and resources (Thom *et al*., 2016; Carter *et al*., 2018; Kokorelias *et al*., 2021).

The health literacy mediator (HLM) role stems from insights described in the Marmot Review: Fair Society, Healthy Lives (Marmot and Bell, 2012). At the time, Marmot suggested that programs involving local health trainers, community champions and community development exhibit promising results in empowering individuals to take control of their own health. The impact on health inequities is uncertain, but this approach encourages individual and community involvement in healthcare, enhances all aspects of HL and supports overall well-being. However, this HLM role we propose here differs from existing positions (e.g. the health navigators and health connectors) by addressing the health inequities that exist in their own specific communities and targeting those who have been disadvantaged by their SDHs. The role would aim to improve all aspects of HL and not just access to healthcare, while actively engaging in health promotion for individuals, organizations and working with policymakers in that local area.

The present research team expanded on this research and explored in further detail the title and role of a HLM, with focus on where this role could be placed and what they can address to have the most impact on health inequities. In a scoping review published in 2021, a HLM was defined as *a person or group of people dedicated to providing learning experiences and opportunities to enable individuals and communities to overcome inequities perpetuated by their social determinants and increase their HL assets to improve their health outcomes* (Spencer *et al*., 2021). For a HLM to be accepted by the people within a community and thereby be useful to those community members throughout their life course, it is vital that the community itself is involved in the development of the roles and responsibilities of the position (Batterham *et al*., 2014). Therefore, the aim of this study was to engage with a subset of community members to acquire local stakeholder perspectives on HL and determine the potential of a HLM role in their local community. We wanted to explore the community acceptance and expectations regarding this new and novel role by allowing active participation from the community during the initial development phases.

## METHODS

This study implemented a constructivist epistemology with qualitative research design. Data were thematically analysed in alignment with the phases outlined by Braun and Clarke (2019). This method of analysis was selected as it encompasses the process of evolving themes that are important to the phenomenon of interest, in this case the HLM (Fereday and Muir-Cochrane, 2006).

### Ethics permission statement

This study was approved by the University of Tasmania Research Ethics Committee (Approval Number H0026170). Data collection commenced after ethics approval.

### Participants and procedures

This research was conducted in the small, but diverse, state of Tasmania, Australia. Participants were recruited through a pool of respondents from a previous phase of a larger research project. Participation was voluntary and there was no compensation for participating. The final participants who were interviewed were randomly selected, using convenience sampling from the earlier pool (Liamputtong *et al*., 2016). The aim was to recruit a minimum of 10 participants for semi-structured one-on-one interviews, a number based on other HL interview-based research in the literature (Beauchamp *et al*., 2017; Warland *et al*., 2023).

Qualitative semi-structured online interviews were conducted. The questions were informed by earlier research surveys that examined local HL strengths and challenges. The question guide can be located in the Supplementary Files. Fourteen community members participated in the study. They were nearly all women, with a variety of ages, from differing IRSD areas, well-educated and working, and about half living with a chronic health condition or disability. Further demographic characteristics can be found in Table 1. The interviews enabled the researcher to explore the local community’s understanding, thoughts and attitudes, via a flexible and unintrusive methodology. Simple questions assisted in guiding the interview discussions, and the semi-structured nature of the interview allowed the researcher and the participant greater flexibility whilst investigating and examining the questions of interest (Liamputtong *et al*., 2016). The research team decided to go beyond the original 10 interviews until data saturation was reached (14), meaning that additional data no longer lead to new insights (Braun and Clarke, 2021). The interviews were recorded and then transcribed verbatim using transcription software (Otter.ai).

**Table 1:** Demographic characteristics

Demographic Variables of Participants	Total (*N* = 14)	
	*n*	Percentage
Gender		
Male	1	7
Female	13	93
Age		
18 – 35 (young adult)	2	14
36 – 55 (middle aged)	6	43
56 + (older adult)	6	43
Nationality		
Australian	14	100
IRSD (Index of Relative Socio-Economic Disadvantage)		
1 (most disadvantaged)	3	21.5
2	6	43
3	1	7
4	3	21.5
5 (least disadvantaged)	1	7
Relationship		
Single	4	28
In a relationship	10	72
Household composition		
Living alone	2	14
Living with others	12	86
ATSI (Indigenous/Torres Strait Islander)		
No	14	100
Education Status		
Tertiary	10	72
Pre-tertiary	4	28
Employment status		
Working	14	100
Living with a chronic health condition/disability		
Yes	8	57
No	6	43

### Data analysis

The data analysis strategy used for this qualitative research was thematic analysis (Braun and Clarke, 2019). Thematic analysis enhanced our understanding of how different individuals perceive HL within their communities and what a HLM would look like in their context. The process of thematic analysis, as outlined by Braun and Clark (2019), involves six phases. First, Author 1 familiarized herself with the data (phase one). The process of familiarization involved transcribing the interviews, repeatedly listening to the audio recordings and thoroughly re-reading the transcripts. During this time, key information from each transcript was highlighted and systematically documented in an Excel spreadsheet. Next, she generated the codes (phase two). An inductive approach was used, starting with observing real-world examples, looking for patterns and then developing theories based on those patterns. The coding process was repeated multiple times, focusing on the data while acknowledging that our analysis was influenced by prior knowledge gained from earlier readings on the topic. Upon further exploration of the data, a theme search was conducted by Author 1 where the codes served as building blocks and were grouped and refined to develop preliminary themes (phase three). These initial themes were discussed and checked by Author 2, this collaborative check ensured that the most relevant points were captured and that they addressed the research aims (phases four and five). The next steps included collecting all qualitative responses, revising the original themes via discussions between all authors allowing for refinement and review of the selected themes, and finally defining the themes to be reported (phase six). The themes and indicative quotes have been presented in the results section below, example quotes were chosen due to their concise and exact nature at reflecting one of the defined themes; however, other participants also provided similar responses.

## RESULTS

Local stakeholder perspectives on HL and the potential of a HLM are summarized in Table 2. Analysis of the qualitative data led to the development of three key themes, each with their own sub-themes:

**Table 2:** Themes, sub-themes and example quotes

Themes	Sub-themes	Quotations
**Health empowerment**	Engagement	Health professionals being a normal part of life means that as an adult I am not afraid to reach out and ask for help or engage with different health resources. I feel like health literacy mediators would further strengthen this feeling of empowerment, which would be a fantastic thing **(P 1)**. The reality is people within the dominant Australian culture do not want to think about being sick. So they ignore things, which means that when they do engage with health, it is usually in crisis or it is acute, so they are stressed, which I think really mediates how people experience a health system as well **(P 10)**. Health literacy mediators might also act as ambassadors for health literacy in the community, promoting it as an important and necessary skill (along with literacy, numeracy and digital literacy skills), leading to better outcomes for patients, carers, families and the community **(P 14).**
	Self-advocacy	During school, this I feel like the right time to learn to be health literacy because it is when you are gaining some independence, and you should know how to look after yourself, including on how to be healthy and how to look after your health in this society **(P 3).** I believe having a health literacy mediator for many people in our community would benefit them. They could teach them how to work with our healthcare system and how to stay heathy **(P 6)**. For many people, support with decision-making about how to deal with their health, especially if it may result in loss of quality of life, or which may be life-threatening, would help provide considerable peace of mind, and a sense of safety and security, at a time of need **(P 14).**
**Meeting needs**	Equity	I think the other way to get good help in the health system is to be a good patient. Like if you are a nice patient, you will get looked after well. How different people are brought up and how they perceive and interact with our healthcare system seems to play a big role in their health **(P 10)**. It is about resourcing where it needs to be resource **(P 11)**. It is top–down approach, the change has to happen at the professional level. It is saying, there is these inequalities brought on by their social determinants. So, what are we doing about that? We are not fixing social determinants and we’re not fixing the communicators. The people with the most power are doing the least, and the people with the least power are expected to do the most **(P 12)**.
	Community	I think that the need for health literacy mediator is a sign that the system is broken. There is a place for them, they need to be deeply embedded in community, they need to be there for a long time, and be well resourced. You must have trust and familiarity and are comfortable enough with that person. They have got to be culturally appropriate. And that does not mean just with indigenous people, or migrants or whatever. You can be remarkably obtuse when you go into communities, and you have not been there and you are not like those people. **(P 12)**. Things work out here if they are delivered by the community. If you have got someone from the community, in that role that understood the community, it is very different than someone from way away who just drives in and delivers that role two days a week and then drives out. But I do think it would be well received **(P 13)**.
**Navigation and access**	Healthcare system	Although I am health literate, I would have liked more of a focus on mental and emotional health. This is something that has come up for me later on in life and it kind of took me by surprise. I always knew about anxiety and depression, but I never thought it could happen to me and impact me this way. If more of an emphasis was placed on this when I was at school, it may have helped to alleviate some of the stress and worry of going through it and not waiting so long to seek help **(P 1)**. I have generally lived in urban settings with good access to healthcare. When I lived more remote for 3 years access was much more limited. Health literacy mediators could at least help people to access services they may not be aware of **(P 4)**.
	Health information	If you get kids through school, being able to take responsibility for their health, in the sense that they know how to access support and information, being able to find what they need as they need it and knowing about regular check-ups. And if that is built into kids’ education then it is preventative health **(P 10)**. I believe the assistance of health literacy mediators would be invaluable for patients, carers and other community members who for various reasons (e.g. being very unwell, or with low-level literacy, numeracy and digital literacy skills) are incapable of understanding the complexities of their health issues and the treatments which may be recommended **(P 14)**.
	Digital healthcare	Accessing quality Australian appropriate information on the internet is always useful and makes you feel supported as you decided how to gain assistance if you have a health issue. Some people, like the elderly are finding it very difficult in these times, they do not respond to online health information so a health literacy mediator may be able to assist in this space **(P 8)**. A reliance on the internet disadvantages people who live in more remote or regional areas where internet connections are not stable, and there are difficulties with connectivity. Generally speaking, having more staff who can talk to people in real time would make things easier for most people I think **(P 9)**.

P = participant.

(1) **Health empowerment**

Theme 1 deals with the importance of an individual feeling that they have influence and control over their own health outcomes. This may include their ability to make informed decisions, actively participate and question health-related decisions and develop the skills, knowledge and attitudes necessary to make better health choices.

(2) **Meeting needs**

Theme 2 provides information on how services and organizations can do better to respond to those in the community who need more support. People want resources to be where they are needed and to be available for those who need them the most. Participants also suggested that there is a need for services, interventions, programs and people to be both accepted by the community they are in, and receptive to their specific needs.

(3) **Navigation and access**

Factors that affect navigation and access to health information, health services and digital healthcare can be found in Theme 3. The participants outlined the failings of the current system, and the underutilization of services. They recognize that more could be done from a systems level (policy and procedure) to make healthcare easier to navigate and accessible to all.

During the interviews, participants were also asked to identify where they believed a HLM should have been situated in their life course to make a difference to their HL, or to think of others in their community and identify where the maximum benefit could be achieved. The results from this question were that the majority of participants (65%) thought they should be positioned throughout a person’s life course, with just over half (55%) of this group putting emphasis on wanting a HLM during times of need (e.g. when they had to involve themselves with healthcare). Seven percent of participants stated the most benefit would be if a HLM were situated in locations that focused on the elderly, while another 7% would have preferred a HLM in situations focused on parents. Finally, 21% of participants thought that HLMs should be targeting young people, and that being in locations accessible to our youth would yield the most benefit towards improving HL.

## DISCUSSION

This thematic analysis aimed to examine the thoughts of community members to learn their perspectives on HL and the potential of a HLM role. The analysis identified three main themes that can be considered when adopting a HLM role: (1) Health empowerment, (2) meeting needs and (3) navigation and access. These three themes will now be discussed before the development of the HLM role is considered in more detail.

### Health empowerment

Health empowerment is not a new concept. The WHO (2009) acknowledges the term ‘health empowerment’ and has stated that it hinges on four key elements: understanding roles, acquiring the necessary knowledge to interact with healthcare providers, developing essential skills and being surrounded by a supportive environment. This theme emphasizes the process of developing self-confidence when accessing and navigating healthcare, the recognition of personal and social resources and decision-making for optimal health. It is important that an individual understands that they have influence and control over their own health outcomes and are an active participant in making health-related decisions. In line with previous research, the participants in this study identified that increasing the feeling of empowerment would result in better engagement and self-advocacy with the healthcare system (Shearer, 2009; Hickmann *et al*., 2022). This concept may work for those who have the capacity to engage and to self-advocate, but research shows that members of the community most in need do not possess the assets to be empowered about their health (Andermann, 2016; Ravaghi *et al*., 2023).

While HL and health empowerment are two distinct concepts, their impacts are deeply intertwined. According to the WHO, HL is critical to empowerment as it enables individuals to understand and use information in ways that promote and maintain good health (Crondahl and Eklund Karlsson, 2016). Empowering patients by closing the HL gap can lead to better patient outcomes and more informed decision-making (Palumbo and Annarumma, 2018; Bhattad and Pacifico, 2022). The interview participants identified that HLMs could have a role in empowering individuals. Participant 14 summarized that a HLM could act as an ambassador for HL within the community, promoting it as an important and necessary skill which could then lead to better outcomes for patients, carers, families and the whole community.

### Meeting needs

The current findings suggest that the needs of the participants’ community are not currently being met, in terms of health information and resources, but also in terms of promotional activities to highlight the availability of health services. The approach to meeting these needs must be equitable in nature as the HL of a community is influenced by its specific needs, which can vary based on factors such as demographics, socioeconomic status and availability of healthcare resources. Therefore, understanding and addressing these needs is essential for improving HL (National Academies of Sciences *et al*., 2018). As one interview participant alluded to, it is often the people with the most power in the healthcare system (e.g. policymakers and bureaucrats) who appear to be doing the least and the individuals/families with the least power (community members) who are expected to contribute the most to improve their own health. This needs to be addressed to reduce health inequities and ensure equal access to healthcare resources for all individuals. As indicated in Table 2, participants stressed the importance of community involvement. The need for a role that links the community with healthcare is clear, but participants have highlighted that it must be tailored to support the needs of the local community.

### Navigation and access

In keeping with previous research (e.g. Farmanova *et al*., 2018), interview participants acknowledged the failings of the current system, which has led to underutilization of services, difficulty in navigating and limited access to health information, health services and digital healthcare. HL plays a crucial role in an individual’s ability to access and navigate healthcare and health information. Participant 9 explained their experience of this: *When I was younger*, *and less confident in my own abilities*, *I think that it would have made finding advice and assistance much easier if I had known more about or had better health literacy*. It has been acknowledge previously that those who do not have strong HL assets often experience poorer health outcomes, have difficulty seeking information and struggle to take responsibility for their health (Protheroe *et al*., 2009). A HL responsive system plays a vital role in how people access and navigate healthcare (Trezona *et al*., 2018). By improving HL at the organizational level, healthcare providers can enhance patient communication, promote shared decision-making and create a supportive environment for individuals with varying levels of HL (Protheroe *et al*., 2009; Mor-Anavy *et al*., 2021). This is not a new challenge: interventions such as the previously mentioned Health Navigators, Health Coaches, Health Advocates have been trialled in different settings, and have had some success in overcoming the difficulties that individuals may face when navigating healthcare (Natale-Pereira *et al*., 2011; Boroumand *et al*., 2020; Perlman and Abu Dabrh, 2020). However, these interventions often targeted navigation and access at the individual level and did little to build capacity, change policy or improve procedures at a systems level to make healthcare easier to navigate and accessible to all.

### Developing the HLM role

The Integrated Model of Health Literacy (Sørensen *et al*., 2012) provides a framework that captures they key themes identified in this research (health empowerment, meeting needs and enhancing navigation/access). If the HLM role aligns with the core components of HL as outlined in the model, then it will encompass elements such as knowledge, motivation, disease prevention, health promotion and competency to access, understand, appraise and apply health information to make informed decisions regarding healthcare (Sørensen *et al*., 2012). A HLM would then also highlight the importance of placing one’s health in context, understanding the influencing factors and taking responsibility for personal health, as the model underscores the interaction between an individual’s assets and societal demands in enhancing HL (Sørensen *et al*., 2012). By incorporating elements such as leadership, integration of HL into organizational structures, patient-centred communication and co-creation with stakeholders, the new role would contribute to building a health–literate system that empowers individuals and communities to take charge of their health (Kayser *et al*., 2019; Sørensen *et al*., 2021).

A ‘life-course approach’ could help support the integration of the HLM role. Special attention should be paid to the themes identified from this study. This approach considers the critical stages, transitions and settings where significant differences can be made in promoting health and well-being throughout one’s life (Banati, 2018). Interventions aligned with a life-course approach can consider intergenerational aspects, minimize risk factors, enhance protective factors, strive for health equity and collaborate with individuals, communities and the system. In so doing, such interventions can effectively tackle health disparities and improve health outcomes across populations (Jones *et al*., 2019; United Nations Department of Economic and Social Affairs (UN DESA), 2023). This approach was also supported by the interview participants as over half stated a HLM should be available any time throughout the life course for anyone, whether that be in times of need or not.

The emerging role of a HLM differs from existing roles like Health Navigators, Coaches and Advocates by focusing specifically on creating educational experiences to address health inequities driven by one’s social determinants. Unlike Health Navigators who guide patients through healthcare systems, or Health Coaches and Advocates who focus on individual health management and empowerment, HLMs aim to enhance community-level HL assets (Boroumand *et al*., 2020; Perlman and Abu Dabrh, 2020; Budde *et al*., 2022). They work to systematically overcome barriers in understanding and using health information, to support health promotion and prevention efforts, thereby improving health at a broader community level (Spencer *et al*., 2021). The impact of HLM would be significant in reducing health disparities, improving health outcomes and empowering communities to make informed health decisions.

A HLM could make a major contribution to health promotion activities. Participants suggested that a HLM could engage in community empowerment initiatives, build partnerships and enable communities to gain control over factors that shape their lives. Additionally, developing and implementing health promotion programs aimed at encouraging healthy behaviours and reducing the risk of chronic diseases is crucial in adopting a healthier community and reducing the burden on healthcare systems (Kumar and Preetha, 2012). These programs should address SDH, raise awareness, provide education and make changes to policies, systems and environments to promote healthy choices (Nutbeam *et al*., 2021). Setting-based approaches tailored to specific environments such as schools, workplaces, libraries and residential areas are also important. These approaches empower individuals and communities by addressing priority health problems within their contexts (Kumar and Preetha, 2012).

Furthermore, it is essential to advocate for and contribute to the development of healthy public policies that foster equity and supportive environments for health. It is important to provide support and training to organizations and individuals involved in healthcare to ensure that they have the necessary HL skill sets to effectively communicate with the community (Nutbeam and Lloyd, 2021). This is where the emerging role of a HLM could make a difference. Data from this study suggested that a HLM would need to focus on capacity-building at the individual, community, system and organizational level. Someone in a HLM role could facilitate HL interventions that aim to raise awareness among healthcare providers, identify patient needs, improve communication strategies and empower patients to enhance their HL skills. In turn, this would mean that the HL of the whole population has the potential to be improved. Also, by using health promotion to educate and assist those in senior positions to implement patient-centred communication protocols and strategies, healthcare organizations could bridge the gap between patients with varying levels of HL and ensure equitable access to care (Levy and Janke, 2016). Done correctly, the emerging HLM role should be able to build capacity of the community in which it is implemented.

Over time, we hypothesize that communities with active HLM programs will show measurable improvements in HL, leading to better health outcomes. As the benefits of HLMs become evident, there may be increased advocacy for policy changes that support the integration of HL initiatives into broader public health strategies. This could lead to more widespread adoption of HLM roles and further systemic improvements. In summary, the implementation of HLMs has the potential to significantly impact both individual health outcomes and broader healthcare systems. By focusing on education and empowerment, HLMs can drive long-term improvements in community health, reduce disparities and contribute to a more equitable and efficient health system. It will be important to determine if these roles already in situ (and just require training to become a HLM) or will this require a new role altogether.

### Strengths and limitations

The current research exhibits notable strengths in its methodology and framework, which are versatile and reproducible across various contexts to facilitate future research. The consistency maintained in interviews, led by a single researcher, enhances the reliability and validity of the study’s qualitative data (Liamputtong *et al*., 2016). Furthermore, this research serves as a crucial component of a larger research project, laying the groundwork for subsequent investigations within the HL field. The findings and recommendations derived from this study are directly applicable to practical settings, particularly on a local scale, thereby aiding in the formulation of policies and practices that are aimed to improve the HL of the population. Finally, the research focuses on an emerging role, thereby contributing novel insights to the field of health literacy.

Limitations to consider include the potential biases that can emerge from qualitative research. It is possible that the interview participants were subject to response bias (saying what they think the researcher wants to hear), since they were drawn from a previous phase of the larger research project and knew the desired outcomes of the project (Morrison *et al*., 2015). To help mitigate the possible effects of response bias, several steps were implemented. First, a level of trust was formed with interview participants and the structure of the discussion was kept as casual as possible to let the conversation flow naturally. When directed questioning was used, the questions were open-ended, and prompts were used to probe for further information. Finally, anonymity was ensured for all participants involved, this was explicitly stated at the commencement of the interview process so that participants could give their honest opinions without fear of being identified (Liamputtong *et al*., 2016; Braun and Clarke, 2019). Afterwards the interview data were analysed by multiple authors, as discussed in the methods, to help mitigate any researcher bias that may have been present. Sample bias was also a possible concern, as those who opted to participate in the interviews shared similar demographic characteristics (the majority were Australian females, who were employed, tertiary educated and either in relationships or living with others) (Morrison *et al*., 2015). Different themes may have emerged if the participants had represented a more demographic profile. However, as highlighted elsewhere in the literature (Fitzgerald *et al*., 2019; Grewenig *et al*., 2023) sample bias in surveys is often unavoidable when utilizing online recruitment techniques. Future research could look at utilizing other recruitment techniques, such as utilizing community notice boards or community events, situating a researcher on the ground in areas of high foot traffic, or offering incentives for participation to include a more diverse participant pool. In future efforts, potential ethical issues would need to be carefully considered.

### Implications for future research

This research allows us to start thinking about community-driven recommendations to address specific HL needs. From our interviews, our recommendations for the emerging HLM role are that this role must focus on:

- Community empowerment initiatives.
- Community health assessments.
- Capacity-building at the individual, community, system and organizational level.

Furthermore, the role itself should contain elements of:

- Flexibility in its setting, so that it can be tailored to specific environments so that priority health problems are addressed.
- Availability, so that it is accessible any time throughout the life course for everyone, whether that be in times of need or not.

Future research should build upon these finding and continue to work with local community members to refine and shape a community-focused, fit-for purpose role of a HLM.

## CONCLUSION

For those that experience challenges with HL, it can be associated with poor health outcomes and be exacerbated by various social and economic determinants (Marmot and Bell, 2012; Sørensen *et al*., 2012). It is vital that those working in the health promotion space adapt novel and innovative ways to improve HL on both a local and on an international scale. This study engaged with local community members to acquire their perspective on the potential of a HLM role. They identified that to accept this emerging role it would need to encompass the attributes of health empowerment and meeting community needs, and to address the existing barriers in navigating and accessing the healthcare system.

## Supplementary Material

daae130_suppl_Supplementary_Files

## Data Availability

The data that support the findings of this study are available on request from the corresponding author. The data are not publicly available due to privacy or ethical restrictions.
