# Supplementary material for: Community voices in health literacy: a qualitative exploration into perceptions of a health literacy mediator
Source: Health Promot Int. 2024 Oct 14;39(5):daae130. doi: 10.1093/heapro/daae130 (PMC11471997; doi:10.1093/heapro/daae130)
Supplement: daae130_suppl_Supplementary_Files [file daae130_suppl_supplementary_files.docx]

# **Supplementary material:** Interview Questions

1. Tell me something about yourself. (Age, occupation, educational attainment, marital status, postcode, pregnancy status, children age, etc. – **prompting used as required**)
2. What challenges/barriers have you experienced in the last 12 months when utilising health information, healthcare, and community health services?
3. Can you think of anything that would help you overcome each of these barriers?
4. How would you describe health literacy in your own words, what does it mean to you (in your world)?
5. Have you heard of health navigators/health coaches/health literacy mediator?

A health literacy mediator is an emerging role and is described as:

*“*A person or group of people dedicated to providing learning experiences and opportunities to enable individuals and communities to overcome inequities perpetuated by their social determinants and increase their HL assets to improve their health outcomes.”

How would they have impacted on your situation?

1. How has your medical history and your upbringing influenced how you access/do not access the healthcare system?
2. Do you believe a health literacy mediator could address this?
3. Where in your life course would you perceive the greatest benefit of a health literacy mediator to be?
4. Evidence suggests that teaching health literacy earlier in the life course (e.g. during childhood) is more beneficial than providing it later in life.

- How much did you learn about your health and how to look after your health at school?
- Would receiving health education earlier have made a difference. Why/ why not?
